# A machine learning algorithm for electrocardiographic fQRS quantification validated on multi-center data

**DOI:** 10.1038/s41598-022-10452-0

**Published:** 2022-04-26

**Authors:** Amalia Villa, Bert Vandenberk, Tuomas Kenttä, Sebastian Ingelaere, Heikki V Huikuri, Markus Zabel, Tim Friede, Christian Sticherling, Anton Tuinenburg, Marek Malik, Sabine Van Huffel, Rik Willems, Carolina Varon

**Affiliations:** 1 Department of Electrical Engineering (ESAT), STADIUS Center for Dynamical Systems, Signal Processing and Data Analytics, KU Leuven, Leuven Belgium; 2 Department of Cardiovascular Diseases, Experimental Cardiology, KU Leuven, Leuven Belgium; 3 Department of Cardiac Sciences, Libin Cardiovascular Institute, Cumming School of Medicine, University of Calgary, Calgary, Alberta Canada; 4 Research Unit of Internal Medicine, Medical Research Center Oulu, University of Oulu and Oulu University Hospital, Oulu, Finland; 5 Department of Cardiology and Pneumology, Heart Center, University of Göttingen Medical Center, Göttingen, Germany; 6 Department of Medical Statistics, University Medical Center Göttingen, Göttingen, Germany; 7 DZHK (German Center of Cardiovascular Research), partner site Göttingen, Göttingen, Germany; 8 Division of Cardiology, University of Basel Hospital, Basel, Switzerland; 9 Department of Cardiology, University Medical Center Utrecht, Utrecht, Netherlands; 10 National Heart and Lung Institute, Imperial College, London, UK; 11 Department of Internal Medicine and Cardiology, Masaryk University, Brno, Czech Republic; 12 Microgravity Research Center, Université Libre de Bruxelles, Brussels, Belgium

**Keywords:** Biomedical engineering, Cardiology

## Abstract

Fragmented QRS (fQRS) is an electrocardiographic (ECG) marker of myocardial conduction abnormality, characterized by additional notches in the QRS complex. The presence of fQRS has been associated with an increased risk of all-cause mortality and arrhythmia in patients with cardiovascular disease. However, current binary visual analysis is prone to intra- and inter-observer variability and different definitions are problematic in clinical practice. Therefore, objective quantification of fQRS is needed and could further improve risk stratification of these patients. We present an automated method for fQRS detection and quantification. First, a novel robust QRS complex segmentation strategy is proposed, which combines multi-lead information and excludes abnormal heartbeats automatically. Afterwards extracted features, based on variational mode decomposition (VMD), phase-rectified signal averaging (PRSA) and the number of baseline-crossings of the ECG, were used to train a machine learning classifier (Support Vector Machine) to discriminate fragmented from non-fragmented ECG-traces using multi-center data and combining different fQRS criteria used in clinical settings. The best model was trained on the combination of two independent previously annotated datasets and, compared to these visual fQRS annotations, achieved Kappa scores of 0.68 and 0.44, respectively. We also show that the algorithm might be used in both regular sinus rhythm and irregular beats during atrial fibrillation. These results demonstrate that the proposed approach could be relevant for clinical practice by objectively assessing and quantifying fQRS. The study sets the path for further clinical application of the developed automated fQRS algorithm.

## Introduction

The presence of fragmentation in the QRS complex (fQRS) on a 12-lead electrocardiogram (ECG) has been reported to be an indicator of myocardial scarring or fibrosis^1^. fQRS has been shown to be related to arrhythmic and mortality risk in patients who received an implantable cardioverter-defibrillator (ICD) in primary prevention of sudden cardiac death^2^. Therefore, fQRS is a biomarker of interest in risk stratification of cardiac patients. Currently, the presence of fQRS is diagnosed by visual interpretation of the 12-lead ECG to identify irregularities within the QRS complex. This requires specific training to distinguish fragmentation from other patterns, such as early repolarization or non-specific conduction delays. Different definitions of fragmentation, which vary slightly in some of the morphologies considered, have been described. The most frequently used criteria are these proposed by Das et al.^1,3^, which define morphologies which should be identified as fQRS, both for narrow ($\le$120 ms) and broad (>120 ms) QRS complexes. However, the application of these criteria might be ambiguous and might lead to intra- and inter-observer variability^4^. Therefore, other criteria have been proposed to overcome these uncertainties, such as those described by Torigoe et al., 2012, where risk stratification was based on the number of fragmented leads^5^, or by Maheshwari et al., 2013, where an automatic approach is proposed to detect different patterns of fQRS^6^. However, these methods have neither been validated nor do they express the severity of fQRS. More recently, Haukilahti et al. defined benign and malignant fQRS variants in an attempt to improve the specificity of fQRS as a biomarker^7^.

As the application of different fQRS criteria and inter-observer variability limit the reproducibility of clinical studies in different centers^8^, the detection of fQRS may benefit from an automatic signal-based, algorithmic approach, to provide decision support. However, there is no true “golden standard” and automatic algorithms trained using predefined fQRS criteria might reproduce the same ambiguities as human annotations. Some automatic fQRS detection algorithms have previously been described most of which are based on signal decomposition methods, such as wavelet transform or intrinsic time-scale decomposition for the delineation of the QRS complex and detection of fragmentation^6,9,10^. However, all these algorithms were trained based on their own fQRS criteria in single-center data. The most recent automatic algorithm published by our group (Goovaerts et al.^11^), was based on the Das criteria for fQRS detection^11^. This model, developed in a single-center cohort of sinus rhythm ECGs including narrow and broad QRS complexes, offers a continuous scoring output between 0 and 1 for fQRS quantification and is based on Variational Mode Decomposition (VMD)^12^ and Phase-Rectified Signal Averaging (PRSA)^13^. Although this algorithm does not distinguish different fQRS patterns, the output was associated with agreement between observers and the risk of all-cause mortality^14^.

This work proposes a fully automated algorithm for fQRS detection and quantification in order to avoid subjective interpretation. In line with this objective, three main contributions are proposed. First, we present a novel and robust QRS complex segmentation strategy which combines multi-lead information and automatically excludes abnormal heartbeats. Valid segmentation of the QRS complex plays a major role in further analysis since too restrictive segmentation might ignore fQRS patterns in the Q or S waves and the inclusion of artefacts or small oscillations might be confused with fragmentation. In previous work, this process has often been based on a single ECG lead, ignoring the complementary information available in multi-lead recordings. Second, the proposed machine learning algorithm for fQRS quantification is trained and evaluated in multi-center data, using different fQRS criteria comprising narrow and broad QRS complexes. Finally, to the best of our knowledge, this is the first evaluation of such an approach on non-sinus rhythm data, extending the applicability also to atrial fibrillation signals (AF). These models were tested and their performance is presented in comparison with visual fQRS detection.

## Methods

**Figure 1:**
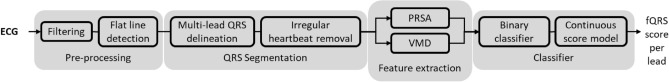
Block diagram of the fQRS scoring algorithm per lead.

The block diagram in Fig. 1 illustrates the methodology proposed. The algorithm uses all leads for segmentation, but feature extraction and classification are applied to each lead independently. As in the Das criteria for region specific fQRS analysis, all leads, except aVR, were used in the analysis^2,3^. Hence, the output presents 11 fQRS scores per patient’s recording. In the remainder of this paper, each lead will be referred to as *signal*, and the set of 11-lead ECG will be referred to as *recording*.

### Data

Two independent datasets were used in this study. The first dataset includes 723 digital ECG recordings (10 seconds 12-lead ECG, 250 Hz sampling rate) of patients before ICD implantation in prevention of sudden cardiac death at the University Hospitals Leuven (UZL) in Leuven, Belgium. For this analysis, only recordings in sinus rhythm and AF were included, corresponding to 673 recordings (616 sinus rhythm and 57 AF). The details of these patients are summarized in Table 1.

The presence of fQRS was annotated independently by 5 clinicians^4^ (referred here as observers), who assigned a binary label to each of the 11 signals as fragmented (i.e. 1) or non-fragmented (i.e. 0) by using the fQRS criteria for narrow and wide QRS complexes defined by Das et al.^1,3^. Each signal in the database was assigned the sum of the independent labels given by the 5 observers, resulting in a joint label per lead with values ranging between 0 and 5 (Table 2). While labels 0 and 5 represent full agreement of the clinicians in the absence or presence of fQRS, respectively, the labels in-between represent inter-observer variability. The signals with full agreement were used as ground truth to train the algorithm, as these signals represent the highest level of certainty.

**Table 1:** Patient characterization for UZL and EU-CERT datasets. Expressed as number of ECG recordings and percentage between brackets, or mean ± standard deviation.

	UZL	EU-CERT
**Demographics**		
Age at implant (y)	62.4 ± 11.6	63.8 ± 10.9
Male gender (%)	570 (84.7)	1006 (79.8)
Ischemic cardiomyopathy (%)	446 (66.3)	869 (68.9)
Left ventricular ejection fraction (%)	32.3 ± 12.3	24.9 ± 6.2
Primary prevention (%)	381 (56.6)	1261 (100)
Sinus rhythm (%)	616 (91.5)	1102 (87.4)
Atrial fibrillation (%)	57 (8.5)	159 (12.6)
**Comorbidity**		
Diabetes mellitus (%)	119 (17.7)	342 (27.1)
Stroke (%)	67 (10.0)	132 (10.5)
**ECG**		
Wide QRS (%)	370 (55.0)	496 (39.3)
Heart rate (bpm)	64 ± 13	72 ± 15
**Follow-up**		
Duration (y)	3.7 ± 3.4	3 ± 2.3
Appropriate Shock (%)	222 (33.0)	165 (13.1)
Death (%)	186 (27.6)	230 (18.2)

The second dataset was provided by the multi-center EUropean Comparative Effectiveness Research to Assess the Use of Primary ProphylacTic Implantable Cardioverter Defibrillators (EU-CERT-ICD) project^15^. This project included a retrospective registry of 5111 patients who received a primary prevention ICD between 2002 and 2014 in one of the 14 participating centers^16^. The cohort presented is based on the detailed ECG analysis reported by Pelli et al.^17^. After further exclusion of rhythms other than sinus and AF, a total of 1560 ECGs were included in the current analysis. This selection contains 402 12-lead recordings from University Hospital Basel, Switzerland (10 seconds, 500Hz, least significant bit resolution of $4\mu \hbox {V}$ provided by C.S); 29 from Oulu University Hospital, Finland (8 seconds, 1000Hz, $2.5\mu \hbox {V}$, by T.K, H.V.H); 551 from University Medical Center Utrecht, Netherlands (10 seconds, 500Hz, $4.88\mu \hbox {V}$, by A.T); 277 from University Medical Center Goettingen, Germany (10 seconds, 500Hz, $4\mu \hbox {V}$ by T.F, M.Z); and 301 from the UZ Leuven, Belgium (10 seconds, 500Hz, $4.88\mu \hbox {V}$, by B.V, R.W). Since the ECGs from UZ Leuven are also part of the UZL data, these were excluded from the EU-CERT-ICD database, reducing it to 1259 ECGs. The details of this dataset are described in Table 1.

As described by Pelli et al.^17^, the presence of fQRS was annotated independently by two clinicians, who later discussed a final binary label for leads at which they previously disagreed. The narrow QRS complexes were annotated using the criteria defined by Haukilahti et al.^7^, while wide QRS complexes were annotated following Das et al.^3^. The binary label 1 of EU-CERT-ICD indicates agreement on the presence of fQRS, and 0 agreement on the absence of fQRS (Table 2). Since this dataset does not contain uncertain labels, the complete set of labels are considered ground truth.

**Table 2:** Percentage of leads per label in the UZL and EU-CERT datasets, both for sinus and AF files.

		Percentage files per label in UZL					
		0	1	2	3	4	5
UZL	sinus	40.95	13.19	7.23	5.76	8.16	24.70
	AF	41.15	13.56	10.85	5.90	6.06	22.49
EU-CERT	sinus	76.60					23.40
	AF	77.70					22.30

The inter-observer variability between UZL and EU-CERT-ICD labels was assessed by comparing the annotations of the 301 recordings shared by both datasets. This comparison was performed by binarizing the UZL labels for different thresholds from 1 to 5, and deriving the Kappa score $\kappa$, for each of these scenarios. The highest agreement ($\kappa$ = 0.46) was obtained when all observers agreed on the presence of fQRS. The relaxation of this threshold by including cases in which fewer observers agreed on fQRS in the positive class implied a linear decrease of $\kappa$. Overall, there was only moderate agreement when using the different clinical criteria to assess fQRS. The criteria for fQRS scoring used in the EU-CERT-project were stricter than the Das-criteria used in the UZL-dataset. This could be expected since all signs of fragmentation were scored in UZL, while in EU-CERT-ICD some notches in the QRS complex were not considered as fQRS unless they represented one of the specific patterns described by Haukilahti et al.^7^.

### Pre-processing

A two-step pre-processing was applied. First, the signals were band-pass filtered using Butterworth filters of orders 4 and 6, respectively from 0.5 to 70 Hz, to remove baseline wander and high-frequency noise while maintaining the potential fQRS information. Second, each signal was normalized by removing the mean and dividing by the standard deviation of all voltage values.

### QRS segmentation

Segmentation is crucial to properly estimate the level of fragmentation within a QRS complex. While it must be wide enough to capture the information in the Q and S waves, it needs to be robust enough not to confuse these waves with oscillations and noise around the QRS complex.

The first step of a robust QRS segmentation is detecting the R-peaks in the ECG signal, which is followed by identification of the surrounding Q and S waves. In this work, R-peak detection was performed using the algorithm included in the R-DECO software^18^. Since this algorithm treats every lead independently, the multi-lead information is integrated in a consecutive post-processing stage. The starting point is the number of heartbeats $n_R$ in the recording, which should be equal in all signals from a same recording. The value $n_R$ is defined as the most commonly found number of automatically detected R-peaks in all signals of one recording. The aim is to have a set ${\mathbf {R}}$ of R-peaks with $n_R$ annotations per signal *l*. If more than $n_R$ R-peaks are detected in a signal, only those $n_R$ complexes closest to the average location of each $R_i$ peak are kept. Conversely, in a given signal $l_i$ in which less than $n_R$ R-peaks are automatically detected, a second search is performed to detect the missed heartbeats by performing a refined maximum search in a window of 40ms in the absolute values of the signal $l_i$ around the average position of the missed R-peak. The pseudo-code for this step is summarized in Algorithm 1. 
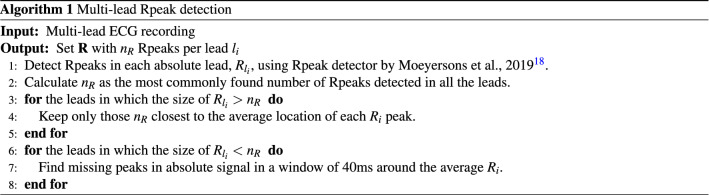


**Figure 2:**
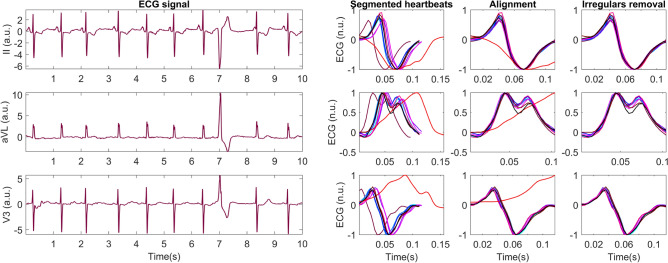
Example QRS segmentation for multi-lead signal with irregular heartbeats, showing the segmentation steps for leads II, aVL and V3. From left to right, the columns show the ECG signal, the segmented heartbeats (step 4 of Algorithm 2), the aligned heartbeats (step 5), and the final segmented heartbeats after removing irregular heartbeats.

After having identified the set ${\mathbf {R}}$ with the same number $n_R$ of R-peaks in all leads, the QRS complexes were segmented. In a review by Beraza et al.^19^, the segmentation method proposed by Martinez et al., 2004^20^, and used in the ECGkit software tool^21^, was identified as the most accurate. This method is based on wavelets and was used to detect the onset and offset of the QRS complex for each lead independently. Next, similar as for R-peak detection, the multi-lead information is integrated in a post-processing stage presented in Algorithm 2. Once the QRS complexes are automatically delineated for each signal, the Q and S waves are reallocated per heartbeat to the median of all signals. Thereupon, these locations are adjusted using the information contained in the rest of the heartbeats of the same signal exploiting the periodicity of the signal. Hence, a set of rules is performed per signal. First, the QRS complexes are segmented and normalized to a maximum amplitude of 1. Next, a template is selected per signal, defined as the QRS complex with the highest cumulative correlation to the other QRS complexes within the lead. The rest of the QRS complexes are aligned to this template, and their Q and S locations are adjusted accordingly. Subsequently, a quality-check is performed to remove irregular and erroneously delineated heartbeats. A vector $\rho = [\rho _1, \rho _2, \dots , \rho _{n_R}]$ is calculated per heartbeat, containing the normalized correlations between it and the remaining heartbeats in the given signal. This vector has $n_R$ elements and compares the given QRS complex to each of the $n_R$ heartbeats in the signal. If more than half of the entries in vector $\rho$ are lower than a quality limit *q*, where $q \in [0,1]$, the given heartbeat is removed from further signal analysis. While *q* can be user selected, it was set here to 0.85 based on visual inspection by an experienced clinician. An example of the main steps of the segmentation strategy for a signal with irregular heartbeats is illustrated in Fig. 2. The QRS segmentation methodology is publicly available together with illustrating demos (https://github.com/avillago/multiLeadSegmentation). 
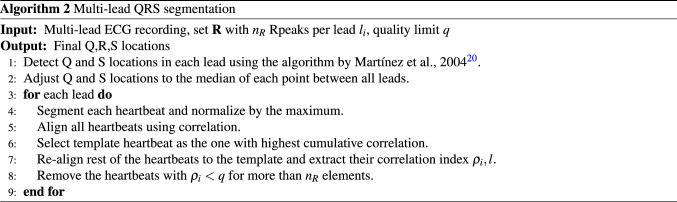

### Feature extraction

The features included in this work are based on the proposal by Goovaerts et al.^11^. The features are extracted independently for each lead, and all the QRS complexes selected in the QRS segmentation stage are considered for feature extraction. The characterization of each signal is based on features extracted from variational mode decomposition (VMD), phase-rectified signal averaging (PRSA) and the number of peaks in the QRS.

**Figure 3:**
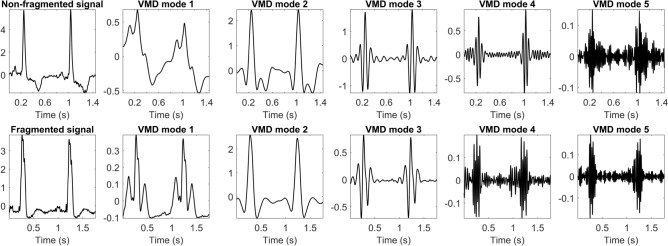
Examples of the VMD decomposition of non-fragmented (upper row) and fragmented (lower row) QRS complexes. Modes 1 to 5 are sorted in increasing frequency.

VMD decomposes the signal into its main frequency components according to their level of contribution to the ECG waves. The number of modes was set to 5 based on previous ECG studies^11,22^, and these were sorted from lower to higher frequency. Since fQRS manifests itself as high-frequency notches in the QRS complex, it was shown that the central frequencies and morphology of the higher-frequency modes were different when comparing fragmented and non-fragmented signals^11^. An example of this decomposition for a fragmented and a non-fragmented signal can be seen in Fig. 3. To capture these differences, the central frequencies and number of zero-crossings of modes 3, 4 and 5 were used as features for classification, since these are the modes that show more differences between both classes.

**Figure 4:**
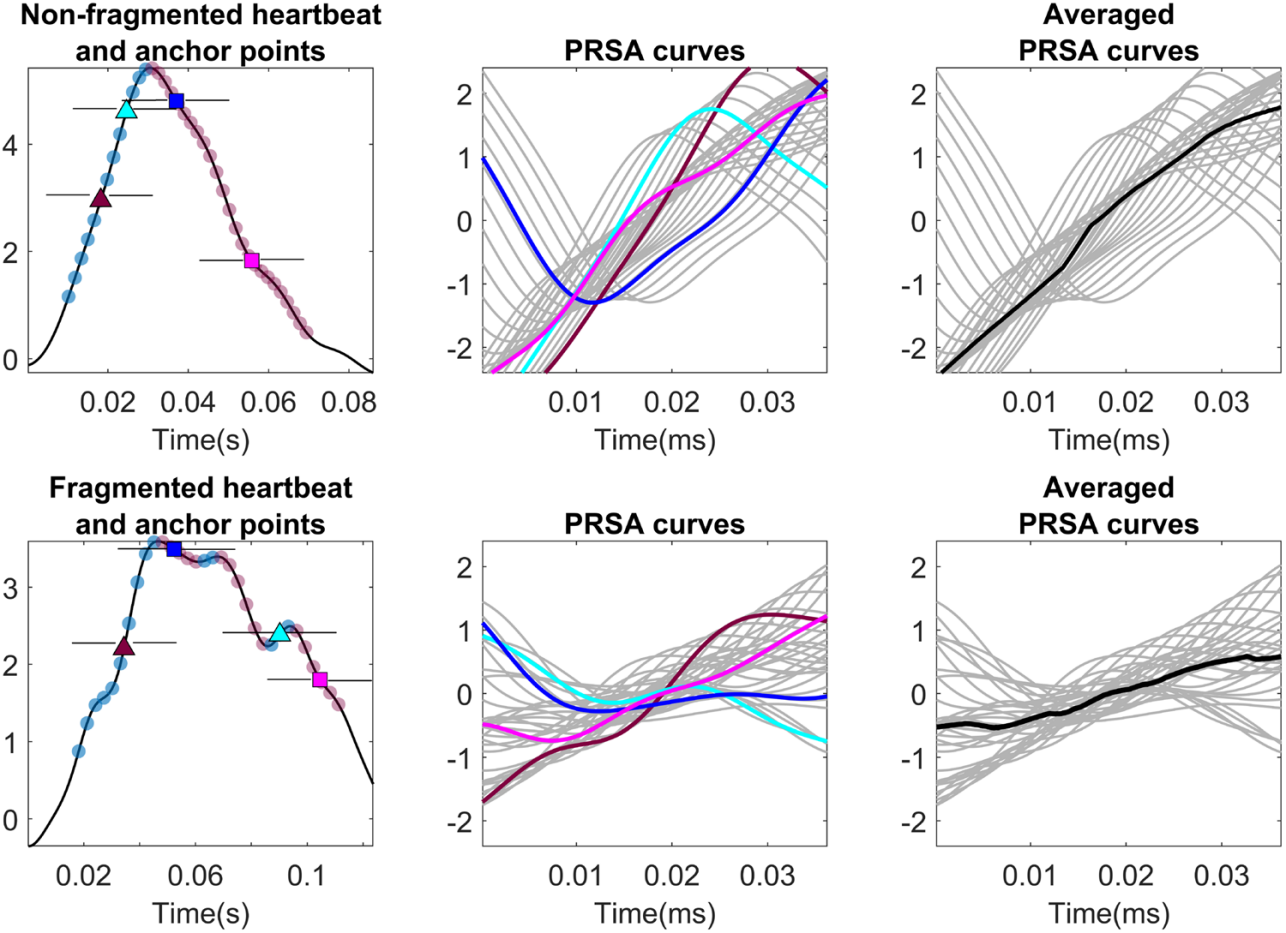
Examples of the PRSA procedure for the analysis of a non-fragmented and a fragmented QRS complex. The first column shows the signal and some examples of the sliding window centers located along the QRS complex. The blue points correspond to samples in increasing segments of the signal, and the pink ones to descending points. The second column shows the PRSA curves extracted around each of these samples, with a length of 40ms. Those corresponding to descending points were inverted so they all follow an comparable trend. The last column shows in black the averaged PRSA curve on top of all the contributing curves.

Additionally, PRSA was used to detect and quantify oscillations in the QRS complex, as disturbances of its expected trajectory. Applying a sliding window of length 2*L*, consecutive overlapping segments of the QRS are averaged out to obtain an estimation of the global trend of the QRS complex, referred to as PRSA curve. This curve can be approximated by a linear fit. Non-fragmented signals present a clear slope in this approximation, characterizing the drastic amplitude change typical of a normal QRS complex. However, the QRS notches in fragmented signals flatten this line, since they disrupt the continuous trend of the QRS complex^11,23^. Some examples of this PRSA methodology are shown in Fig. 4. The features extracted from PRSA analysis are the mean derivative of the averaged PRSA curve and the slope and y-axis crossing of its linear fit approximation. Finally, the number of peaks in the QRS complex are also used as feature to describe the signal. The complete list of features is provided in Table 3.

**Table 3:** List of features extracted from each lead.

VMD features	
Central frequencies	Mode 3
	Mode 4
	Mode 5
	Mode 3
Number of zero crossings	Mode 4
	Mode 5

PRSA features	
Mean derivative PRSA curve	
Slope linear fit	
Y-crossing linear fit	

ECG features	
Number peaks QRS	

### Classifier

These 10 features extracted from each signal were used to train a Support Vector Machine (SVM)^24^, a machine learning classifier, to discriminate between fragmented and non-fragmented signals. Each signal was introduced as an independent input, with no context of other leads^2^. The binary labels indicating the presence of fQRS, as described in Section 2.1, were used as an established baseline to train the classifier. To account for possible non-linearities in the data, SVM with three different kernels were used to transform the feature space, namely the linear, polynomial and radial basis function (RBF). The parameters required to use them were tuned using Bayesian optimization. The SVM classifiers provide a binary output, which corresponds to fragmented or non-fragmented signals. To quantify fQRS, Platt scaling was used to convert the binary output of the model into a continuous score by fitting a logistic regression to the outputs^25^. By doing so, the model extracts the probability of a signal belonging to the fragmentation class.

### Experiments

The different challenges of this study required independent training strategies and experiments to confirm their added value to the final algorithm. The first goal was to evaluate the impact of the new segmentation algorithm on the quantification of the fQRS. Secondly, we wanted to assess the effect of different fQRS definitions on the training and performance of the classifier. Finally, we evaluated the performance of the model in irregular signals form patients in atrial fibrillation (AF).

#### Experiment 1: The impact of the new segmentation algorithm

The impact of the proposed QRS segmentation algorithm was assessed by comparing its performance with the method proposed by Goovaerts et al.^11^, referred to as the *reference*. Both approaches were trained and tested on the same subset of the UZL dataset. First, training and evaluation was performed using only those signals in which all 5 observers agreed in the presence or absence of fQRS, i.e. labels 0 and 5. Patients were randomly split into training (80%) and test (20%), referred as *test1*. In order to reduce bias, this random division was performed 10 times, leading to training and evaluation with the reference and the proposed methodology in 10 different training-test sets. Next, the best model for each of these 10 repetitions was tested by adding the signals with labels from 1 to 4, both for the reference and proposed algorithm. This group of signals with annotations between 0 and 5 is referred to as *test2*. It allowed a comparison of the continuous score obtained from the algorithm with the certainty of fragmentation, represented by the agreement among the observers.

#### Experiment 2: The effect of different fQRS annotation criteria

The second experiment aimed at evaluating the effect of different fQRS annotation criteria in the multi-center data on the automated fQRS algorithm. By evaluating the algorithm in both UZL and EU-CERT-ICD datasets, its performance could be assessed from two different perspectives, acknowledging the effect of the different fQRS definitions used. The final goal was to select a model that could be implemented in clinical practice by combining both approaches.

Three different training strategies were used:

1. Training exclusively on UZL. Here, the model selected in Experiment 1 was used, testing it in the corresponding UZL test signals and on the complete EU-CERT-ICD dataset.
2. Training exclusively on 80% of the data from EU-CERT-ICD. Given the lower percentage of fragmented leads in this dataset, a balanced set of signals was used for training the classifier. Therefore, only the same number of fragmented signals present in the training set were randomly selected from all the non-fragmented signals of the training patients. The other 20% of the EU-CERT-ICD patients were used for testing, as well as the complete UZL dataset.
3. The third strategy merged both datasets, using 80% of each dataset for training, while the remaining 20% of each was used as an independent test set. Similar to the previous strategies, out of these 80% of the patients, only the training leads labeled 0 or 5 were used in UZL, and a balanced set of leads was selected for EU-CERT-ICD.

**Figure 5:**
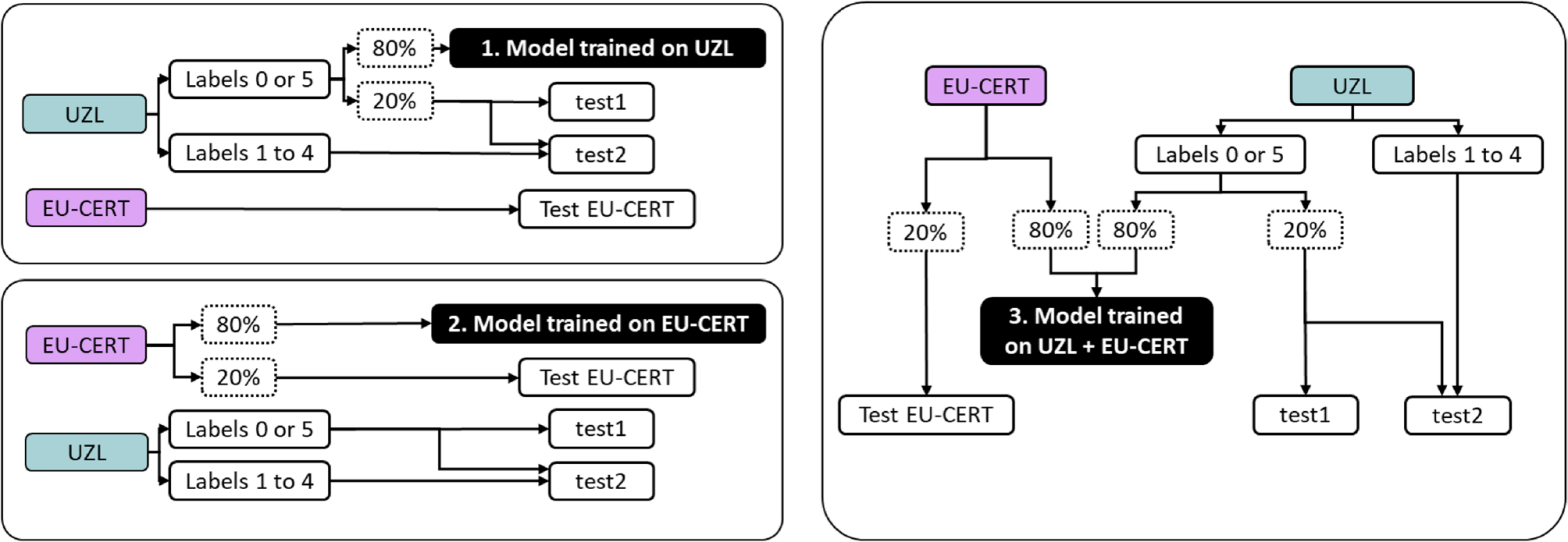
Block diagram describing the training strategies to evaluate the effect of multiple fQRS annotation criteria.

A summary of the data assignment is shown in Fig. 5. Given the random selections used to assign signals to the training set, each of these divisions was performed 10 times to reduce bias, and the set leading to the best results was kept in each case.

#### Experiment 3: Using the fQRS algorithm in irregular AF recordings

All experiments previously described were trained and evaluated on ECGs with regular sinus rhythm. Due to the morphology similarities of QRS complexes in sinus and AF signals, the introduced models should also apply to AF signals. In previous works, these signals were excluded from analysis due to their irregularities, since the short interval between heartbeats in AF might pose a problem to this type of algorithms. In the last experiment the best model selected from Experiment 2 was applied to the AF recordings of UZL and EU-CERT-ICD to assess the performance of the algorithm in ECGs with an irregular rhythm.

### Performance metrics

The models were evaluated in test sets with different characteristics. Due to the lack of a “gold standard” for fQRS annotation, the signals scored 0 or 5 from UZL and the binary labels from EU-CERT-ICD based on 2 observers are considered as the established baseline. Therefore, the results were evaluated using sensitivity, specificity, positive predictive value (Sens, Spec and PPV, respectively) and area under the curve (AUC) both for the receiver operator characteristic (ROC) and precision-recall (PR) curves. The latter is particularly relevant in the experiments that involve the EU-CERT-ICD dataset, given that its labels are highly imbalanced with fewer fragmented signals. In such a situation, the PR AUC provides more informative results than the ROC AUC^26^. These metrics were derived by binarizing the algorithm fQRS score using a 0.5 threshold. In the cases in which 10 iterations for each of the 3 kernels proposed were performed, the results of these 6 metrics are provided as the mean and the standard deviation. Additionally, Kappa scores ($\kappa$), reflecting the agreement between the algorithm scores and the manual labelling, are presented. The $\kappa$ scores were calculated several times for each experiment, exploring 100 different thresholds uniformly distributed between 0 and 1. The reported $\kappa$ values correspond to the highest score obtained during these iterations. Lastly, the models were also evaluated on the UZL signals where observers did not agree. Since these signals involve uncertainty, the Pearson correlation coefficient between the labels and the continuous output of the models was calculated.

### Analysis of feature importance

Once the best model from Experiment 2 was selected, the contribution of the features to the model output was explored performing a feature relevance analysis. This analysis was performed using SHapley Additive exPLanations (SHAP), which is a methodology based on game theory to explain the outputs of machine learning models and improve their interpretability^27,28^. For every feature, the SHAP value quantifies the contribution of the future to the final prediction of the signal as fragmented or not fragmented. Positive SHAP values indicate that the feature positions the signal towards the fragmented class, and negative values towards the non-fragmented class.

### Ethical approval and informed consent

The study was conducted in accordance with the Declaration of Helsinki and Good Clinical Practice principles. The UZ Leuven study was approved by the ethical committee of the University Hospitals of Leuven with registration number S56074. The EU-CERT-ICD study was an international collaboration approved by all local ethics committees and was registered on ClinicalTrials.gov (NCT02064192). Given the retrospective design of both cohorts the need for informed consent was waived by the local ethics committees.

## Results

### Experiment 1: The impact of the new segmentation algorithm

**Figure 6:**
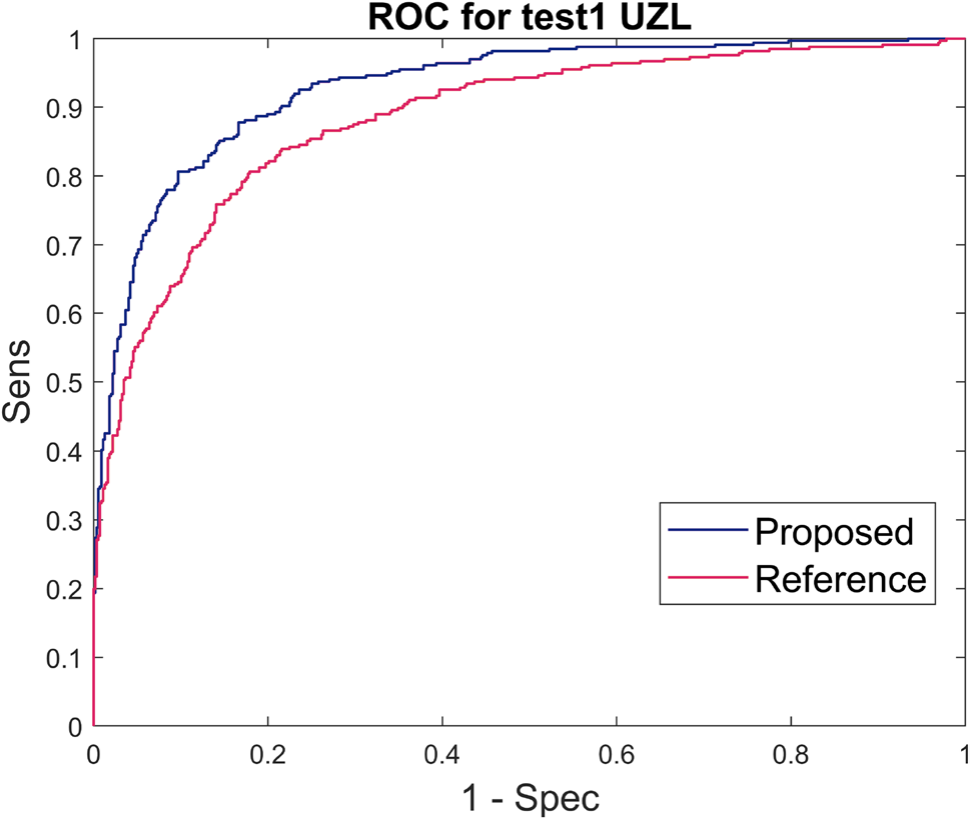
ROC curves for the proposed and reference approaches applied to test1 signals of UZL dataset, exclusively including signals without inter-observer variability.

The sensitivity, specificity, PPV, ROC AUC and $\kappa$ score results for the first experiment are shown in Table 4 and Fig. 6. The first row of each block in Table 4 shows the average and standard deviation of 30 experiments: the 10 training-test combinations for each of the 3 kernels (linear, polynomial and RBF). The difference in results between the 3 kernels were minimal, as can be seen in Table S1 of Supplementary Material, indicating that the feature space allowed a linear separation between both classes. Table S1 presents the breakdown of the results for each of the kernels, as the mean and standard deviation of the 10 training-test combinations. Taking into account the similar results for all kernels, the models selected made use of the linear kernel for simplicity. The best training-test set was selected out of the 10 combinations and the results of the classifier using the linear kernel are shown in the *Selected model* row of each block in Table 4.

The newly proposed approach achieved better results than the reference for all metrics. The PR AUC values were 0.89 and 0.84 for the proposed and reference approaches, respectively. This small difference can be explained by the high percentage of fragmented signals in the UZL test set making it almost balanced.

**Table 4:** Results of the proposed and reference approach on test1 of UZL including only signals with agreed labels between annotators. The first row of each block shows the average and standard deviation of each metric for all the iterations performed (10 training-test combinations for each of the 3 kernels). The second one shows the results of the selected models, corresponding to the best training-test combination for the linear kernel.

Algorithm		Sens	Spec	PPV	ROC AUC	$\kappa$
Proposed	Average + STD	0.72 ± 0.06	0.92 ± 0.01	0.86 ± 0.02	0.93 ± 0.02	0.68 ± 0.04
	Selected model	0.76	0.92	0.86	0.93	0.71
Reference^11^	Average + STD	0.68 ± 0.02	0.89 ± 0.02	0.8 ± 0.02	0.88 ± 0.01	0.62 ± 0.03
	Selected model	0.68	0.89	0.79	0.88	0.62

**Figure 7:**
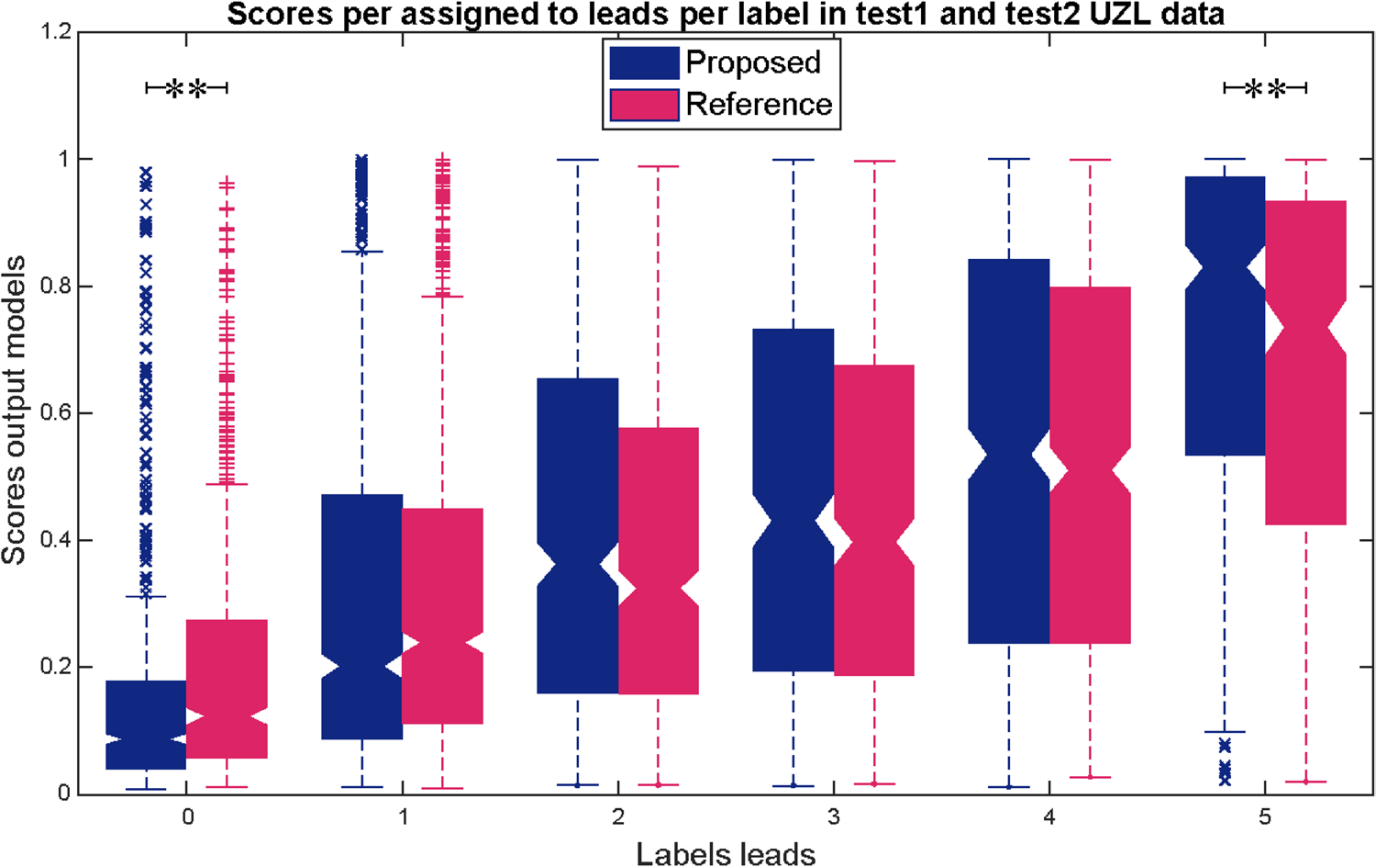
Relation between the output scores of the proposed and reference approaches (in blue and pink) with the labels of test2 UZL signals, ranging from 0 to 5. Friedman test was applied to compare the results of both approaches, obtaining p-values below 0.005. These statistically significant differences are described by the sign ** in groups 0 and 5.

Next, the continuous outputs were evaluated in the complete UZL-dataset with signals with labels ranging from 0 to 5. The boxplots in Fig. 7 show the scores assigned by the proposed (blue) and reference (pink) model to the signals labeled 0 to 5. For both approaches the continuous score increased with the higher labels, illustrating the relation between the automated scores and the inter-observer variability. Pearson correlation coefficients were 0.50 for the proposed approach, and 0.45 for the reference (both p < 0.001). Additionally, in the proposed model, the scores assigned to the 0-labelled and 5-labelled signals were more skewed towards 0 and 1 values, respectively, which is reflected in the average scores for each of these labels presented in Table 5. The scores assigned by each of the approaches to these two labels were statistically significantly different both for 0-labelled and 5-labelled. Therefore, we can conclude that compared to the reference algorithm, the proposed approach provides more consistent scores for the signals with inter-observer agreement.

**Table 5:** Summary of the results of the proposed and reference approach in test2. The first two columns show the average ± standard deviation fQRS scores assigned to signals labelled as 0 and 5. The third and forth columns show the correlation coefficients between the fQRS scores and the ground truth labels from 0 to 5, both with p-values below .001.

	0	5	Pearson Corr.	p-value	95% CI
Proposed	0.16 ± 0.2	0.72 ± 0.29	0.50	$\ll$ .001	[0.48, 0.53]
Reference^11^	0.2 ± 0.21	0.65 ± 0.3	0.45	$\ll$ .001	[0.42, 0.47]

### Experiment 2: The effect of different fQRS annotation criteria

**Figure 8:**
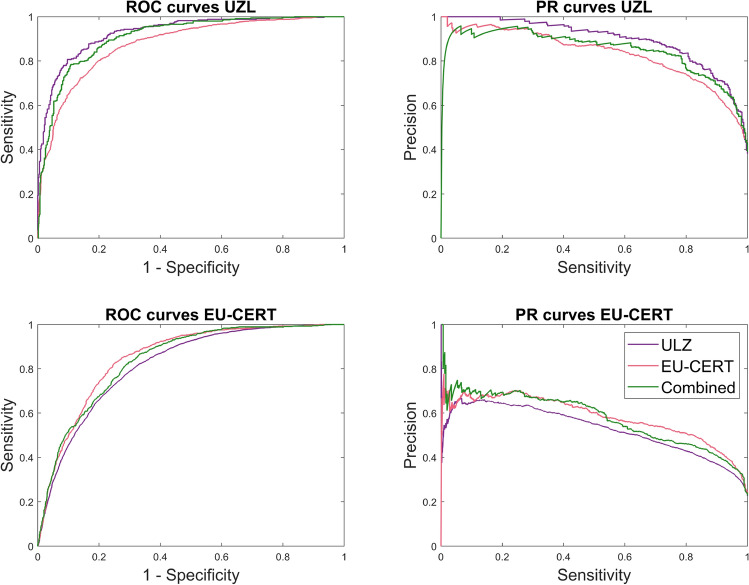
ROC and PR curves for test1 UZL and EU-CERT for the 3 training strategies considered.

The results of experiment 2 are shown in Table  6 and Fig. 8. For UZL test1 signals, the ROC and PR curves were very similar, whereas for EU-CERT-ICD, the PR AUC was lower than the ROC AUC. As expected, the best performance was obtained when the test set belonged to the same center as the data that was used to train the model. The trade-off achieved by the combined model was of interest since the performance for UZL was only mildly reduced whereas the performance in the EU-CERT-ICD test improved when compared to the EU-CERT-ICD trained approach. These variations might be attributed to the lower percentage of fragmented signals and stricter fQRS criteria in EU-CERT-ICD. This was confirmed by the $\kappa$ scores since the best results on the EU-CERT-ICD data were achieved for thresholds of 0.5 for the model trained on the same dataset, and for thresholds greater than 0.8 for the models that included UZL data in the training stage. On the other hand, the thresholds that led to the best results in UZL were lower than 0.5 (0.42, 0.31 and 0.47 for training strategies 1, 2 and 3, respectively).

**Table 6:** Results of the 3 training strategies to evaluate the effect of multiple fQRS annotation criteria. From top to bottom, each row shows the results for the approach trained on UZL, on EU-CERT, and in both. The left side of the table shows the results for test1 UZL data, and the one on the right for EU-CERT.

Training	test = UZL						test = EU-CERT					
Strategy	Sens	Spec	PPV	ROC AUC	PR AUC	$\kappa$	Sens	Spec	PPV	ROC AUC	PR AUC	$\kappa$
UZL	0.76	0.92	0.86	0.93	0.89	0.71	0.90	0.56	0.37	0.82	0.53	0.41
	0.74 ± 0.02	0.92 ± 0.01	0.84 ± 0.02	0.92 ± 0.01	0.86 ± 0.02	0.69 ± 0.02	0.90 ± 0.00	0.56 ± 0.01	0.37 ± 0.00	0.82 ± 0.00	0.53 ± 0.00	0.41 ± 0.00
EU-CERT	0.59	0.92	0.84	0.88	0.83	0.61	0.77	0.78	0.51	0.85	0.58	0.48
	0.57 ± 0.02	0.92 ± 0.00	0.84 ± 0.01	0.87 ± 0.00	0.83 ± 0.00	0.59 ± 0.02	0.74 ± 0.03	0.77 ± 0.01	0.48 ± 0.02	0.83 ± 0.01	0.55± 0.02	0.46 ± 0.01
UZL +	0.75	0.90	0.83	0.91	0.85	0.68	0.88	0.64	0.42	0.84	0.57	0.44
EU-CERT	0.73 ± 0.02	0.90 ± 0.02	0.83 ± 0.01	0.90 ± 0.01	0.84 ± 0.02	0.66 ± 0.02	0.86 ± 0.02	0.64 ± 0.01	0.42 ± 0.01	0.83 ± 0.01	0.55 ± 0.03	0.44 ± 0.02

Subsequently, the 3 training strategies were evaluated in UZL test2 signals. The continuous scores obtained for each of the 3 strategies were compared to the labels using Pearson correlation, obtaining coefficients of 0.5, 0.56 and 0.48 (all p < 0.001) for the UZL, EU-CERT-ICD and the combined model, respectively. Table 7 breaks down the results of the combined model by QRS duration both in test1 UZL and EU-CERT-ICD data. There was a lower sensitivity when the algorithm was applied to narrow QRS complexes.

**Table 7:** Results for the combined model in test1 UZL and EU-CERT broken down by QRS duration.

QRS duration	test = UZL						test = EU-CERT					
	Sens	Spec	PPV	ROC AUC	PR AUC	$\kappa$	Sens	Spec	PPV	ROC AUC	PR AUC	$\kappa$
Narrow	0.69	0.91	0.86	0.91	0.87	0.68	0.86	0.68	0.53	0.85	0.67	0.49
Broad	0.83	0.90	0.80	0.92	0.84	0.73	0.90	0.62	0.33	0.84	0.47	0.44

### Experiment 3: Using the fQRS algorithm in irregular AF recordings

Table 8 shows the results of applying the combined model proposed in experiment 2, to binary-labeled signals of UZL and EU-CERT-ICD with AF where the percentage of fQRS labelled signals is 22% and 33% respectively, compared to previously shown performance in regular sinus rhythm signals. As it can be seen, there was a sensitivity decrease in both datasets, accompanied by a slight increase in specificity and PPV. This indicates that while the detected signal fragmentation was generally correct, some AF signals, that were considered to be fragmented on visual inspection, were missed by the model.

**Table 8:** Results of the combined model obtained from Experiment 2 in AF signals of both UZL and EU-CERT.

Test rhythms	test = UZL						test = EU-CERT					
	Sens	Spec	PPV	ROC AUC	PR AUC	$\kappa$	Sens	Spec	PPV	ROC AUC	PR AUC	$\kappa$
AF	0.63	0.96	0.89	0.90	0.86	0.63	0.74	0.73	0.43	0.82	0.54	0.43
sinus	0.75	0.90	0.83	0.91	0.85	0.68	0.88	0.64	0.42	0.84	0.57	0.44

### Analysis of feature importance

**Figure 9:**
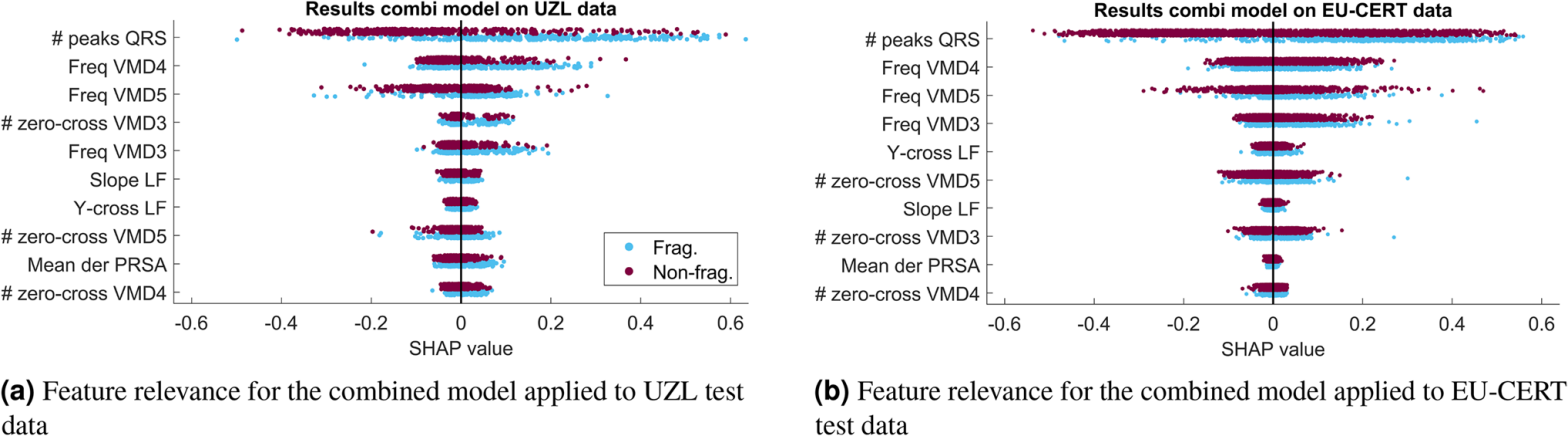
SHAP analysis of UZL and EU-CERT test signals for the combined model.

Figure 9 shows the feature ranking according to their contribution to the final prediction of the combined model, and their influence in each of the test data. While SHAP values for a same feature may vary for different patients, in these figures the features are arranged according to their average SHAP value in order to classify their global importance. The color of the points corresponds to the actual class to which the signals belong: blue and purple points correspond to the fragmented and non-fragmented classes, respectively. As seen, the most important features are the number of peaks in the QRS complex, and the central frequencies of the highest VMD modes. However, this relevance ranking considers feature interaction, even though these interactions are not visible in Figure 9. While the three first most important features are common for both test data, there is a slight change of order from the 4th feature between both groups.

## Discussion

We proposed and validated a new, fully automated machine learning approach for quantification of QRS fragmentation (fQRS) compared to the visual assessment of fQRS by clinical observers. The main contributions of our novel approach are:

- The definition of a new multi-lead algorithm for QRS segmentation, integrating information from different leads for a more robust QRS delineation. This method is publicly available at https://github.com/avillago/multiLeadSegmentation.
- Automatic removal of irregular heartbeats from the fQRS analysis process. This new QRS segmentation strategy includes a method to align the heartbeats in each lead, which allows deriving fQRS results only from normal and atrial premature heartbeats.
- Training and evaluation on a multi-center dataset, including ECG signals from 5 different European hospitals, allowing to assess the impact of inter-observer variability and the use of different fQRS criteria.
- The new algorithm was proven to be usable in both sinus rhythm and atrial fibrillation, as well as in narrow and wide QRS complexes.

The results show that the new QRS segmentation method improved the performance to detect visually confirmed fQRS, when compared to the earlier described reference method. The PPV and specificity indicate that the modified approach reduced the number of false positives. The sensitivity did improve as well. Further, the results indicate a better discrimination of the most evident fQRS cases compared to the reference, since the scores of signals in the UZL-dataset where all observers agreed (i.e. labels 0, as non–fragmented, and 5 as fragmented) were more skewed to the extreme scores 0 and 1^11^.

Currently, the only available comparator for our automated approach is the visual assessment of fQRS. This is not only prone to intra- and inter-observer variability^4^, but different definitions of fragmentation have also been proposed for clinical practice. The impact of different fQRS criteria on the training and performance of our automated fQRS quantification method are of interest. Previous automated approaches were exclusively trained with a single definition of fQRS and involved fewer observers. In our study, the models trained exclusively on one dataset achieved low results when tested on the other one, due to their differences in fQRS criteria. The fQRS criteria used to score the EU-CERT-ICD dataset considered the magnitude and location of the fragmentation. Annotating only specific fQRS patterns and allowing some benign patterns to be labelled as non-fragmented, result in a more restrictive annotation and a lower prevalence of fQRS. The use of these criteria poses a challenge to the proposed algorithm, since the features used do not include information about the location of the notches detected. The VMD and PRSA features suggested by Goovaerts et al. exclusively inform about the presence of fQRS and are extracted for the entire QRS complex, with no information with regards to its location. This can be inferred from the results of the model trained and evaluated on EU-CERT-ICD data. This EU-CERT-oriented model detected several false positive classifications, as indicated by the lower specificity, PPV and the results of the PR-curves, when compared to the UZL-oriented model. The difference in fQRS criteria explains the opposing behavior in terms of sensitivity and specificity for both datasets. While the models in which UZL data was included in the training detect most of the fragmented signals of EU-CERT-ICD, this also resulted in a higher number of false positive classifications in the EU-CERT-ICD data. On the contrary, an exclusively EU-CERT-ICD-oriented approach achieved low values of sensitivity in the UZL data, but the combined strategy did not compromise the performance on the UZL data while offering a trade-off when testing on the EU-CERT-ICD data. Hence, we propose that the combined model would be the most generally applicable approach to clinical practice, combining the different views on fragmentation. While this approach combines different criteria presenting some strengths versus a single-database trained approach, its results are clearly influenced by the annotations used for the training. Therefore, this algorithm does not fully overcome the issue of inter-observer variability. However, in clinical practice the proposed approach could be used as a recommendation system for fQRS analysis. Those cases with more uncertain fragmentation scores, such as those ranging between 0.4 and 0.6, could require additional experienced visual inspection, while scores closer to 0 and 1 might be considered as recommended scores.

Figure 9 shows the relevance of the features in the output of this combined model. The main challenge of this evaluation is the presence of two different fQRS definitions, both for the training and evaluation stages. It can be seen that the feature relevance for UZL and EU-CERT data is slightly different from the fourth relevant feature. One reason for these differences is the large overlap between the fragmented and non-fragmented classes for the EU-CERT signals. This confirms the need of additional features for a better learning of the EU-CERT annotations, which could include characteristics such as the location of the notches in the QRS, as well as the number of notches. However, even the results for UZL do not show large SHAP values for the features related to PRSA. A reason for this could be the relevance of the QRS duration, which might lead to different results for narrow and wide QRS complexes due to the number of samples available (Fig. 4).

**Figure 10:**
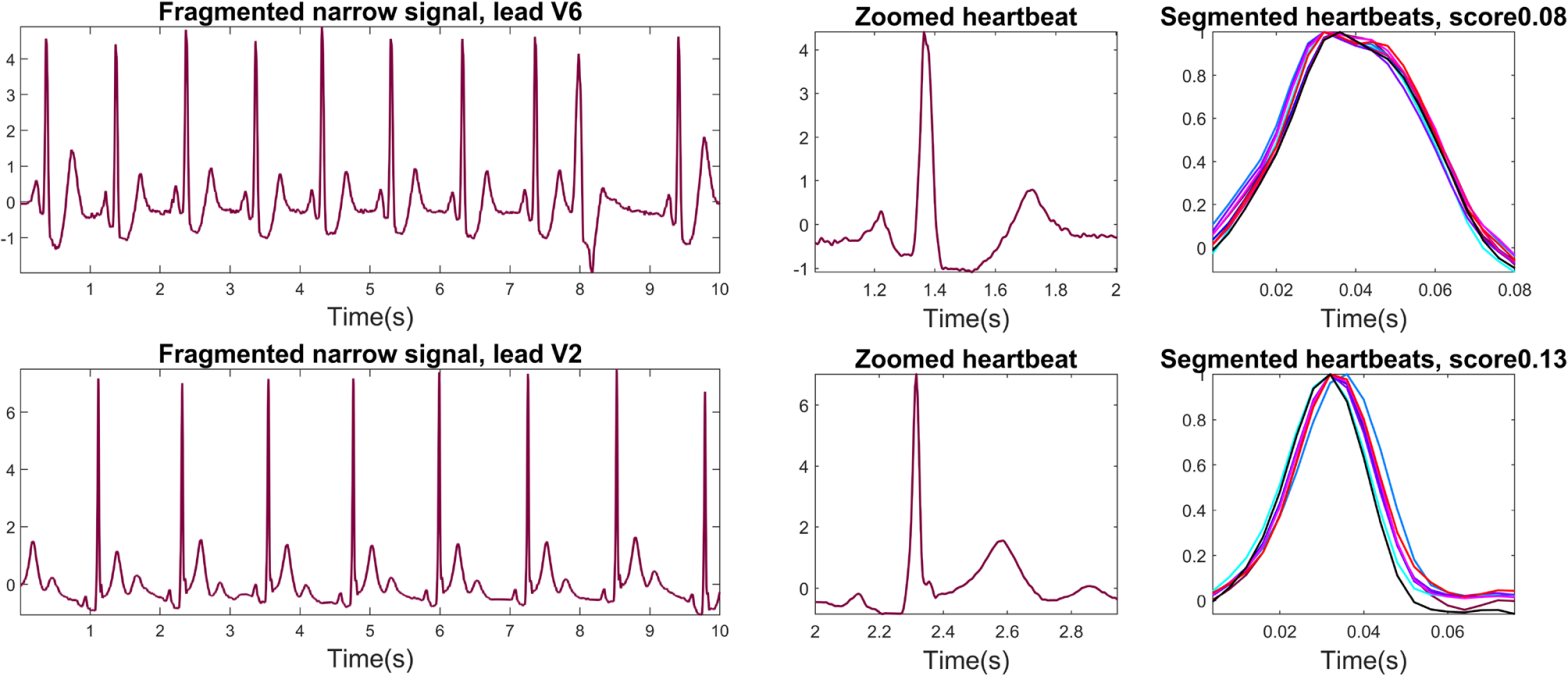
Example of two narrow QRS signals where the labels and the automatic score disagree (low algorithm score for fragmented signals). The top signal shows slight fQRS in the R-peaks, visible in the zoomed heartbeat and in the segmented heartbeats. The second presents fragmentation on the S wave, which lead to errors in QRS delineation and hence on scoring.

The breakdown of results in terms of width of the QRS complex was not reported before for other automated approaches, despite its relevance in the fQRS analysis. Our results show a difference in performance depending on the QRS duration of the signals, especially in sensitivity and PPV in UZL data. The reduced sensitivity in narrow QRS complexes may indicate that the segmentation is more accurate in broad QRS complexes, but also that fragmentation is more evident in these signals. After observation of narrow QRS cases annotated as fQRS but receiving a low score from the algorithm, it was observed that many of these correspond to minimal fragmentation. The example in the upper row in Fig. 10 shows a minimal notch after the R-peak which is annotated as fQRS when applying the criteria by Das et al. However, the automated algorithm calculated a low score and considers this complex non-fragmented. These cases may be missed by the algorithm, particularly in narrow QRS complexes as there may not be enough samples capturing these abnormalities. These cases highlight the relevance of quantifying fQRS. Whether this minimal notching is clinically relevant is unknown and requires further investigation.

The second row of Fig. 10 shows another example of a fragmented signal which received a low fQRS score by the automated algorithm. This is a specially challenging case, since fragmentation is only present in the end of the QRS complex. Additionally, this signal containing narrow QRS complexes and sampled at 250Hz becomes even more arduous, since the segmentation error is reduced to less than 10 samples. These particular types of heartbeats with notches in the Q and S waves may benefit from specific machine learning-based delineation algorithms, trained on representative examples.

Lastly, previous work never evaluated automated fQRS detection and quantification in AF recordings and algorithms were applied exclusively to regular sinus rhythm signals. In our study, the results obtained for these signals are in line with those of narrow sinus signals in UZL data. For EU-CERT-ICD, these results vary more. It should be noted that these results are affected by the low number of AF signals and the low prevalence of fQRS in AF signals.

### Limitations and future directions

In view of the known limitations of visual inspection of fQRS, the main limitation of our work is the training of our machine learning classifier using an imperfect standard. There is also no clinical consensus on the preferred fQRS definition. We tried to avoid this by using only the signals where all 5 observers agreed in the UZL-dataset thus avoiding inter-observer variability and by combining both criteria in the final model.

Since our goal was to technically validate the algorithm for signal analysis, we processed signals of separate ECG leads individually. In future work the obtained score per signal/lead needs to be summarized in a global score and a score per region/combination of leads (anterior (V1-5), lateral (I, aVL, V6) and inferior (II, III, aVF). We compared and validated the model to clinically used fQRS scoring. In the future, this continuous output of the fQRS algorithm could be used in clinical risk stratification by deriving a combined/regional score and correlating it to the clinical endpoints, e.g., severe ventricular arrhythmia or mortality. These regional scores have been shown to capture relevant information related to outcome. Vandenberk et al. showed that the presence of fragmentation in the inferior leads is related to the manifestation of arrhythmia; while fragmentation in the anterior leads is related to mortality^2^. However, both the regional and per-lead fQRS scores considered in that study are sensitive to inter-observer variability and would benefit from a standardized approach to identify fragmentation in order to derive relevant clinical conclusions. Machine learning applications could be useful to overcome the challenge of inter-observer variability and deriving an optimal combination of scores to optimize risk stratification using fQRS. To this end, future work should explore using representation and unsupervised learning techniques to unveil hidden patterns relevant for this task.

## Conclusion

This work proposed an automated machine learning method for fQRS detection and quantification. A novel, publicly available multi-lead QRS segmentation method was presented, combining the information of the multi-lead ECG and the different heartbeats in the signal. After segmentation QRS features were extracted, based on VMD and PRSA, they were used to train a machine learning classifier (Support Vector Machines) to discriminate between fragmented and non-fragmented ECG-traces. Data from 5 centers in which 2 different fQRS criteria were applied, were used in the training and evaluation of this new model. The combined model, which was trained using data from multiple centers and annotated with both fQRS criteria, showed to achieve comparable results to the specific models while making the model more flexible and more generally applicable to multi-center data, bringing it closer to clinical practice. The algorithm was shown to be applicable to narrow and wide QRS complexes and regular and irregular rhythms, such as AF. These findings are crucial for the next steps to apply these machine learning models in clinical practice and assist clinicians in their diagnosis.

## Supplementary Information

Supplementary Information 1.

## Data Availability

The **codes** for multi-lead QRS segmentation are available at https://github.com/avillago/multiLeadSegmentation.
