# Supplementary material for: A machine learning algorithm for electrocardiographic fQRS quantification validated on multi-center data
Source: Sci Rep. 2022 Apr 26;12:6783. doi: 10.1038/s41598-022-10452-0 (PMC9043208; doi:10.1038/s41598-022-10452-0)
Supplement: Supplementary file 1 — Supplementary Information 1. [file 41598_2022_10452_MOESM1_ESM.pdf]

Supplementary material: Table for results of different kernels, Experiment 1.

Table S1. Results for Experiment 1 for each of the 3 kernels considered in the SVM classifier. The results are expressed as mean $\pm$ standard deviation, summarizing the 10 repetitions performed per kernel.

Approach	Kernel	Sens	Spec	PPV	ROC AUC	PR AUC	κ
Proposed	Linear	0.74 ± 0.02	0.92 ± 0.01	0.84 ± 0.02	0.92 ± 0.01	0.86 ± 0.02	0.69 ± 0.02
	Polynomial	0.70 ± 0.09	0.92 ± 0.01	0.84 ± 0.03	0.91 ± 0.03	0.85 ± 0.04	0.67 ± 0.06
	RBF	0.73 ± 0.02	0.92 ± 0.01	0.84 ± 0.02	0.92 ± 0.01	0.87 ± 0.02	0.69 ± 0.02
Reference	Linear	0.67 ± 0.03	0.89 ± 0.02	0.78 ± 0.02	0.88 ± 0.01	0.82 ± 0.02	0.62 ± 0.02
	Polynomial	0.67 ± 0.07	0.90 ± 0.02	0.80 ± 0.02	0.89 ± 0.02	0.83 ± 0.02	0.62 ± 0.04
	RBF	0.69 ± 0.04	0.90 ± 0.02	0.81 ± 0.02	0.88 ± 0.01	0.83 ± 0.02	0.63 ± 0.02

Supplementary material: EU-CERT-ICD Study Investigators

Department of Cardiology, Semmelweis University Heart Center, Budapest/Hungary: Béla Merkely MD, Peter Perge MD, Zoltan Sallo MD, Gabor Szeplaki MD, Nandor Szegedi MD, and Klaudia Vivien Nagy MD; Department of Cardiology and Pneumology, Heart Center, University Medical Center, Göttingen, Germany: Markus Zabel MD, Lars Lüthje MD, Simon Schlögl MD, Rajeeva Sritharan MSc, Helge Haarmann MD, Leonard Bergau MD, Joachim Seegers MD, Gerd Hasenfuß MD, Pascal Munoz-Exposito MD, Tobias Tichelbäcker MD, and Aleksandra Kirova MD; DZHK (German Center for Cardiovascular Research), partner site Göttingen, Göttingen, Germany: Gerd Hasenfuß MD, Tim Friede MD, Markus Zabel MD, and Simon Schlögl MD; Department of Medical Statistics, University Medical Center Göttingen, Göttingen, Germany: Tim Friede PhD and Markus Harden, PhD; National Heart and Lung Institute, Imperial College, London, United Kingdom: Marek Malik MD, and Katerina Hnatkova PhD; Department of Medical Physiology, University Medical Center Utrecht: Marc A. Vos PhD; Institute for Social Medicine, Epidemiology and Health Economics, Charité Universitätsmedizin Berlin, Berlin, Germany: Stefan N. Willich MD and Thomas Reinhold PhD; University Hospitals of Leuven, Leuven, Belgium: Rik Willems MD and Bert Vandenberk MD; Magdalena Klinika, Department of Cardiology, Krapinske Toplice, Croatia: Janko Szavits-Nossan MD and L. Rotkvić MD; Attikon University Hospital, 2nd Department of Cardiology, Athens, Greece: Panayota Flevari MD, Andreas Katsimardos MD, and Dimitrios Katsaras MD; Slovak Medical University NUSCH, Bratislava, Slovakia: Robert Hatala MD and Martin Svetlosak MD; Medical University of Lodz (MUL) WAM Hospital, Department of Cardiology, Lodz, Poland: Andrzej Lubinski MD and Tomasz Kuczejko MD; Gentofte Hospital, Copenhagen, Denmark: Jim Hansen MD; University Hospital, Department of Cardiology, Basel, Switzerland: Christian Sticherling MD and David Conen MD; KBC Sestre Milosrdnice, Department of Cardiology, Zagreb, Croatia: Nikola Pavlović MD, Šime Manola MD, Ozren Vinter MD, and Ivica Benko RN; University Medical Center Utrecht, Department of Cardiology/Physiology: Anton Tuinenburg, MD, David Sprenkeler, MD, Agnieszka Smoczynska, MD, and Marc A. Vos PhD; University Hospital Tübingen, Department of Cardiology: Axel Bauer MD, Christine Meyer-Zürn MD, and Christian Eick MD; Rigshospitalet, The Heart Centre, Department of Cardiology, Copenhagen University Hospital, and Department of Clinical Medicine, University of Copenhagen, Copenhagen, Denmark: Jesper Hastrup Svendsen MD; IDIBAPS, Department of Cardiology, Hospital Clinic Barcelona, Spain: Josep Brugada MD and Elena Arbelo MD; SUSSCH, Department of Cardiology, Banska Bystrica, Slovakia: Gabriela Kaliska MD and Jozef Martinek PhD; Technische Universität München, Med. Klinik und Poliklinik I, Klinikum rechts der Isar, Munich, Germany: Georg Schmidt MD, Michael Dommasch MD, and Alexander Steger MD; Klinikum Großhadern, Department of Cardiology, Ludwig-Maximilians-Universität Munich, Germany: Stefan Kääb MD, Axel Bauer MD, Moritz F. Sinner MD, Konstantinos D Rizas MD, and Wolfgang Hamm MD; Acibadem City Clinic Tokuda Hospital, Department of Cardiology: Vassil Traykov MD; Medical University of Lodz (MUL), CKD Hospital, Department of Cardiology, Lodz, Poland: Iwona Cygankiewicz MD, Pawel Ptaszyński MD, K. Kaczmarek MD, and I. Poddebska MD; St. Ekaterina University Hospital, Department of Cardiology, Sofia, Bulgaria: Svetoslav Iovev MD; University Hospital Brno, Department of Internal Medicine and Cardiology, Brno, Czech Republic: Tomáš Novotný MD and Milan Kozak MD; Oulu University Hospital and University of Oulu, Medical Research Center, Finland: Heikki Huikuri MD, Tuomas Kenttä MD, and Ari Pelli MSc; Bieganski Hospital, Chair and Department of Cardiology, Medical University of Lodz (MUL), Lodz, Poland: Jaroslaw D. Kasprzak MD and Dariusz Qavoq MD; KBC Rijeka, Department of cardiovascular disease, Rijeka, Croatia: Sandro Brusich MD, Ervin Avdovic MD, and Marina Klasan RN; University Hospital, Department of Cardiology, Olomouc, Czech Republic: Jan Galuszka MD, and Milos Taborsky MD; St. Anna Hospital, Department of Cardiology, Sofia, Bulgaria: Vasil Velchev MD; Klinikum Reinkenheide Bremerhaven, Department of Cardiology, Germany: Rüdiger Dissmann MD; National Heart Hospital, Department of Cardiology, Sofia, Bulgaria:Tchavdar Shalganov MD; Poznan Medical University, HSUH Hospital Department of Cardiology, Poznan, Poland: P. Guzik, T. Krauze; Marienkrankenhaus Bonn, Department of Cardiology, Germany: Dieter Bimmel MD and Christiane Lieberz RN; Klinikum Ludwigsburg, Department of Cardiology, Germany: Stefan Stefanow MD, Norman Rüb MD, and Christian Wolpert MD; University Hospital Regensburg, Department of Cardiology, Germany: Joachim Seegers MD and Lars S. Meier MD; Vivantes Humboldt Klinikum Berlin, Department of Cardiology: Steffen Behrens MD; KBC Split, Department of Cardiology, Croatia: Zrinka Jurisic; Karolinska Institutet, Department of Cardiology, Stockholm, Sweden: Frieder Braunschweig MD; Charité Campus Virchow Klinikum, Department of Cardiology, Berlin, Germany: Florian Blaschke MD and Burkert Pieske MD; General Hospital Zadar, Croatia: Zoran Bakotic MD and Ante Anic MD; Klinikum Weiden, Department of Cardiology, Germany: Robert H.G. Schwinger MD; Lund University Hospital, Department of Cardiology, Lund, Sweden: Pyotr Platonov MD.
